# Morphological Characteristics of Renal Artery and Kidney in Rats

**DOI:** 10.1155/2014/468982

**Published:** 2014-03-04

**Authors:** Atilla Yoldas, Mustafa Orhun Dayan

**Affiliations:** ^1^Adana Veterinary Control and Research Institute, 01150 Adana, Turkey; ^2^Department of Anatomy, Faculty of Veterinary Medicine, University of Selçuk, 42031 Konya, Turkey

## Abstract

The gross anatomy and morphometry of the kidney and renal arteries were studied in the strains of laboratory rat: Sprague-Dawley (Sp) and Wistar (W) rats. Total of 106 three-dimensional endocasts of the intrarenal arteries of kidney that were prepared using standard injection-corrosion techniques were examined. A single renal artery was observed in 100% of the cases. The renal arteries were divided into a dorsal and a ventral branch. The dorsal and ventral branches were divided into two branches, the cranial and caudal branch. Renal arteries were classified into types I and II, depending on the cranial and caudal branches and their made of branching. The present study also showed that the right kidney was slightly heavier than the left one and that the kidney of the male was generally larger than that of the female. The mean live weights of the Sprague-Dawley and Wistar rats were found to be 258.26 ± 5.9 and 182.4 ± 19.05 g, respectively. The kidney weights were significantly correlated (*P* < 0.01) with body weights. The kidney weights were not found significantly correlated (*P* > 0.01) with the length of renal arteries.

## 1. Introduction

In mammals, the right and the left renal artery supply kidneys originate from the related side of the abdominal aorta. They give rise to the dorsal and ventral branches before entering the hilus of the kidney [1–4]. The dorsal and ventral branches, respectively, were divided into the interlobar, arcuate, and interlobular arteries [5]. In some species [2, 6, 7], each kidney is thought of as supplied by a single renal artery. However, some anatomic studies show that two or more renal arteries can supply a kidney [3, 8–11]. Variations in renal vascular anatomy have been important in increasing frequency of experimental renal transplantation, vascular reconstruction for congenital and acquired lesions, and abdominal aortic aneurysms [12]. Furthermore, morphometric parameters of kidney can change in different environmental conditions [13]. For these reasons, it is anatomically and clinically important to bring out the differences and similarities between the rat and other animals.

Although the morphology of the renal artery has been well documented in dog [3, 11, 14, 15], pig [16, 17] sheep [4, 18], cat [2, 6], rabbit [19], calve [20], and human [7, 10, 21–23], there was just one study about the rat kidney arterial anatomy [2]. On the other hand, the renal arteries of human [1, 24], monkey [25], and dog [11] were also classified by taking account of their renal arteries branching, and the renal arteries of the SD and W were not classified.

The aim of this present study is to investigate the morphometric parameters of kidney and the renal arteries of two rat strains. In addition, this study will contribute to the anatomic literature and identify the similarities and differences of other mammalian.

## 2. Material Methods

28 Wistar (14 males, 14 females) and 28 Sprague-Dawley (14 males and 14 females) healthy rats, with the average of 12 months old, obtained from the animal house of Cukurova University at Adana, Turkey, were used for the present study.

The colony was maintained under temperature (20 ± 1°C), relative humidity (50–80%), and illumination (12 h light, 12 h dark) controlled conditions room. The animals were nourished standard rat feed (procured by Feeding Company, Tavas Ltd., Turkey) and ad libitum water. All experimental procedures were approved by Adana Veterinary Control and Research Institute's Animal Experiments Local Ethics Committee (Approval number 28.10.2010/10).

The rats were weighed alive using a weighing balance (EB-3200H balances, Shimadzu Corp., Japan) with sensitivity of ±0.01 g before they were sacrificed.

At first, they were anaesthetized with combination of 10 mg/kg xylazine (Rompun enj; Bayer Turk Kimya San. Ltd. Sti., Istanbul) and 100 mg/kg ketamine HCl (Ketalar Eczacıbaşı, Istanbul) intraperitoneally. Heparin (Liquemine IV, Roche Mustahzarları San. A.S.) was administered (450 U/20 g, IV) slowly to prevent coagulation. Secondly, the animals were euthanasiad by bled by means of an incision to the aorta ascendens, while they were in deep anesthesia; the vessels were washed with 0.9% physiological saline. The kidneys were obtained along with the renal arteries, weighted using Mettler balance with a sensitivity of ±0.01 g. Then, takilon (20% powder monomethyl methacrylate and 80% liquid polymethyl methacrylate) was injected into the renal artery. The originally described corrosion cast method was applied to the materials [26]. Just after injecting takilon, materials were kept at room temperature for 24 hours for polymerization. Finally, they were corrosion-casted in 30% KOH at 60°C for 24–48 hours, washed with tap water, and photographed.

The Nomina Anatomica Veterinaria [27] was used for the terminology.

The arteries were measured with a digital calipers (DV892 calipers, Tecnotest Corp., Germany) with sensitivity of 0.1% mm.

All recorded weights and lengths were expressed as mean ± SEM (standard error of mean) and subjected to statistical analysis using Statistical Package for Social Sciences (SPSS, Predictive Analytics Solutions, London, UK) version 17.0. Paired sample *t*-test at 95% confidence interval was used to determine the level of significant difference in values between sexes. Values of *P* < 0.05 were considered significant.

## 3. Results

### 3.1. Renal Arteries

**Figure 1:**
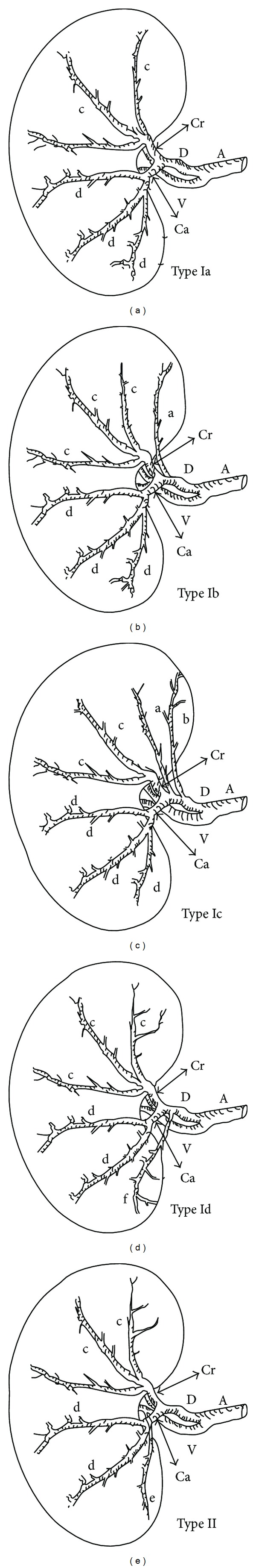
Schematic drawings of arteries of rat kidneys, illustrating the branching patterns of the different types of either the dorsal or ventral divisions of the renal artery. A: renal artery; V: the ventral branch of renal artery; D: the dorsal branch of renal artery; Cr: the cranial division of the renal artery; Ca: the caudal division of the renal artery; a: first interlobar branch from the renal artery for the cranial pole; b: second interlobar branch from the renal artery for the cranial pole; c: interlobar branches from the cranial division of the renal artery; d: interlobar branches from the caudal division of the renal artery; e: extrainterlobar branch from the renal artery for the opposite pole; f: extrainterlobar branch from the renal artery for the caudal pole.

**Figure 2:**
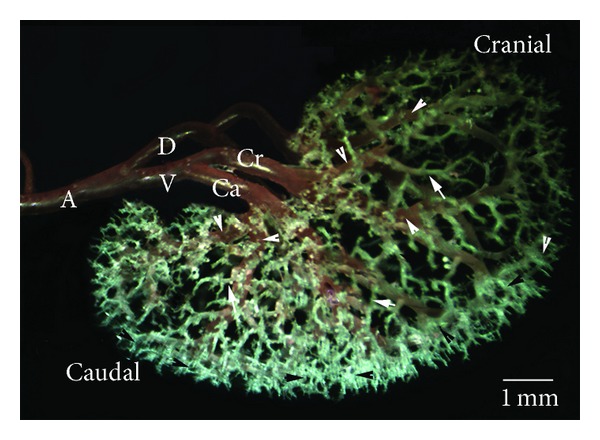
Ventral view of the intrarenal branches of the renal arteries, Type Ia. A: renal artery; V: the ventral branch of renal artery; D: the dorsal branch of renal artery; Cr: the cranial division of the renal artery; Ca: the caudal division of the renal artery; white arrowhead: interlobar arteries; arrow: arcuate artery, black arrowhead; interlobular arteries.

In both strains, the right and left renal arteries were seen to be emerged from the abdominal aorta. The left renal artery originated from lateral aspect of abdominal aorta at just origin of mesenteric cranial artery. It was shown that the right renal artery arose slightly cranial to the left renal arteries, from the lateral side of the abdominal aorta at the 0.5–0.6 cm caudal to celiac artery, whereas in two W (7.1%) and one SD (3.5%), the left and right renal arteries arose from the abdominal aorta at the same level. In all cases, after its origin, the renal arteries were divided into two primary divisions as a dorsal (D) and a ventral branch (V). In two rat strains, we observed that primary divisions were divided into two secondary branches (segmental arteries), as cranial and caudal branches. Segmental branches gave off 5–7 interlobar arteries. It was also seen that the dorsal or ventral branches gave off one or two extra branches in 43 of W's kidneys and 41 of SD's kidneys. The interlobar arteries were observed to give off several arcuate arteries (Figures 1 and 2). The arcuate arteries were spread over the entire surface of the kidney. The interlobular arteries originated from arcuate arteries. No significant anastomoses were seen macroscopically between any of the subbranches of the renal arteries.

### 3.2. Classification of Renal Arteries

The dorsal and ventral branches (primary branches) of renal artery were classified as Type I and Type II due to their origin, supplied area, and also whether they have extra branch or not. The percentages of these types were given at Table 3.

Type I was divided into four subtypes according to the occurrence of small extra branches of either the dorsal or ventral divisions of the renal artery directed to the renal poles.

*Type Ia*. Extra branches were absent. The primary branch had just been only divided into cranial and caudal branches (Figures 1 and 2). The cranial and caudal branches of dorsal and ventral divisions had the same size of forming a balanced circulation. But, in 2 cases of the W, caudal branches of the dorsal division of the left renal arteries were more dominant than cranial branches and also they supplied caudal zone and a part of dorsal mid zone kidneys.

**Figure 3:**
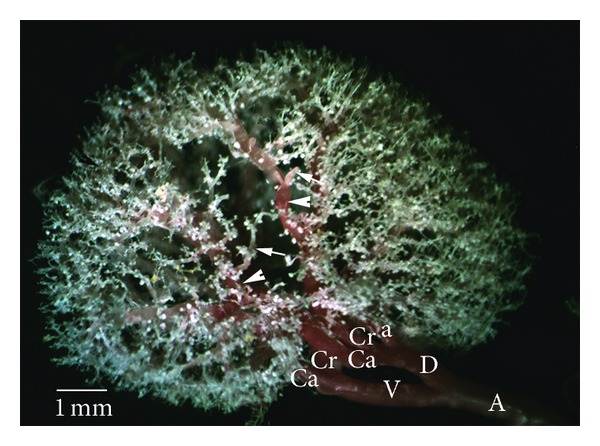
Dorsal view of the intrarenal branches of the renal arteries, Type Ib. A: renal artery: V: the ventral branch of renal artery; D: the dorsal branch of renal artery; Cr: the cranial division of the renal artery; Ca: the caudal division of the renal artery; a: first interlobar branch from the renal artery for the cranial pole; white arrowhead; interlobar arteries.

*Type Ib.* The ventral or dorsal division, after its origin, gave off an extra branch to cranial pole (Figures 1 and 3). In this type, the big part of cranial pole was supplied by an extra branch. Moreover, the cranial branch of dorsal or ventral division was fed to dorsal mid zone and ventral mid zone, respectively. But, in two of SD kidneys and one of W kidneys, dorsal mid zone was supplied by the caudal branch of ventral division and dorsal division. The subtype was the most occurring in the cases (Table 3).

**Figure 4:**
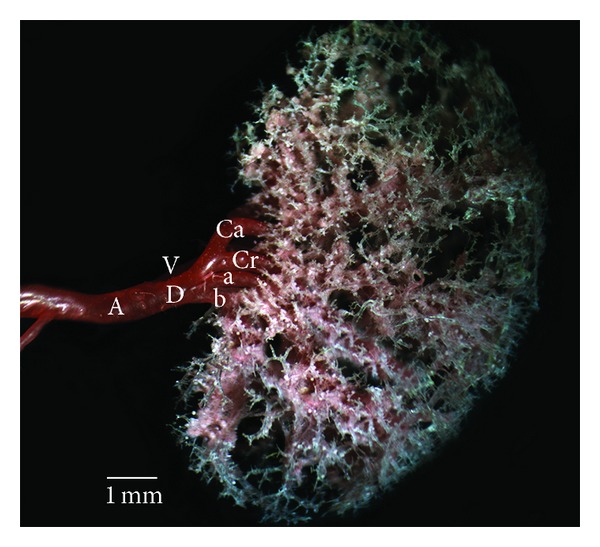
Dorsal view of the intrarenal branches of the renal arteries, Type Ic. A: renal artery; V: the ventral branch of renal artery; D: the dorsal branch of renal artery; Cr: the cranial division of the renal artery; Ca: the caudal division of the renal artery; a: first interlobar branch from the renal artery for the cranial pole; b: second interlobar branch from the renal artery for the cranial pole.

*Type Ic.* The dorsal or ventral division sent two extra branches to cranial pole which was supplied by extra branches. The dorsal mid zone and ventral mid zone were fed by cranial branch of both dorsal and ventral division (Figures 1 and 4). The subtype was just only found in the one SD kidney and two W kidneys (Table 3).

**Figure 5:**
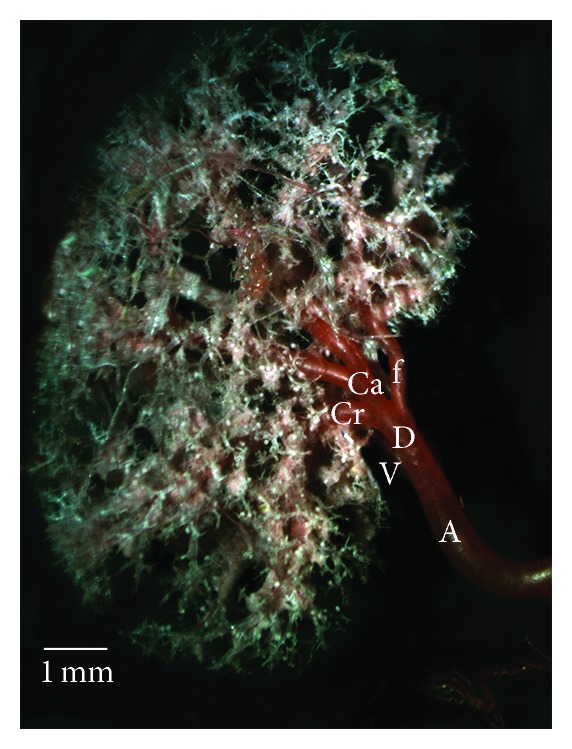
Dorsal view of the intrarenal branches of the renal arteries, Type Id. A: renal artery; V: the ventral branch of renal artery; D: the dorsal branch of renal artery; Cr: the cranial division of the renal artery; Ca: the caudal division of the renal artery; f: extrainterlobar branch from the renal artery for the caudal pole.

*Type Id.* The dorsal or ventral division gave off an extra branch to caudal pole (Figures 1 and 5). In this type, caudal pole was supplied by an extra branch. It was seen that the caudal branch of both dorsal and ventral division irrigated the dorsal mid zone and ventral mid zone. In a kidney of W, the cranial branch was seen to be giving off some branches to mid dorsal zone.

**Figure 6:**
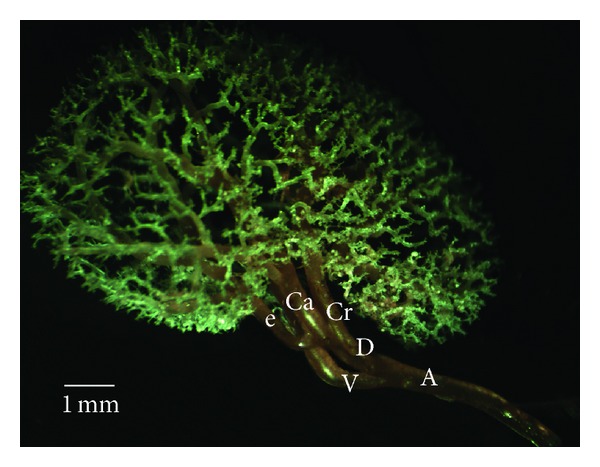
Dorsocaudal view of the intrarenal branches of the renal arteries, Type II. A: renal artery; V: the ventral branch of renal artery; D: the dorsal branch of renal artery; Cr: the cranial division of the renal artery; Ca: the caudal division of the renal artery; e: extrainterlobar branch from the renal artery for the opposite pole.

*Type II.* The ventral division sent a branch to the opposite of the dorsal surface or dorsal division sent a branch to opposite of the ventral surface. The least frequent branching pattern was Type II in the materials (Table 3). In all materials, this branch was separated to the cranial or caudal pole and supplied part of kidney's pole. There was also an interlobar artery in one of the left kidneys SD, originated from the dorsal branch. It separated the ventral mid zone (Figures 1 and 6).

### 3.3. Findings of Statistical Analysis

The mean live body weight of W and SD was 182.4 ± 19.05 and 258.26 ± 5.9 g, respectively. In both strains, mean body weight of male rat was heavier than that of female rat. Mean right kidney weight was heavier than the left one (Table 1). On the other hand, the kidney weights were significantly correlated (*P* < 0.01) with body weights.

The distance between the start point of renal arteries and the division point of dorsal and ventral branches was measured in two strains. In W and SD, this lentgh of right renal arteries (A1) is longer than left ones (A2). But, the length of dorsal (D2) and ventral (V2) branches of left renal artery was longer dorsal (D1) and ventral (V1) branches of right ones. While a consistent positive correlation (*P* < 0.01) was found between live weight and A (length of the renal artery before giving the dorsal and ventral branches), nonsignificant (*P* > 0.05) difference was obtained between live weight and D, V (the length of dorsal and ventral branches of renal arteries). On the other hand, significant (*P* < 0.05) difference was found between kidney weight and D, V.

The ventral and dorsal branches gave off about 5–7 interlobar arteries. There was no positive correlation found between the body weight of rat and the number of interlobar branches (Table 2). Furthermore, it was seen that the number of interlobar branches of renal arteries was not statistically different in kidneys of male and female.

In 89.3% [25] of right kidneys of W and 71.4% [20] of right kidneys of SD, it was shown that ventral branch was stronger than dorsal branch according to its interlobar arteries. But, in left kidneys, this range was 75% [21] in W, 64.3%, in SD [18].

## 4. Discussion

The mean live weight of the W and SD in this study (182.4 ± 19.05 and 258.26 ± 5.9) was within the range (180–250 g) mentioned by Rytand [28] while that of the W (182.4 ± 19.05 g) was lower than the values of some species of rats [13, 28, 29]. Moreover, the mean live weight of the Wistar was higher than range of Onyeanusi et al. [13].

The present study also showed that the right kidney was slightly heavier than the left kidneys and the kidney weights were significantly correlated (*P* < 0.01) with body weights in two strains. Onyeanusi et al. [13] have also similar findings in their study.

As reported by authors [2, 5], we observed that in 100% in SD and in 98.3% of W the renal arteries originated from each side of abdominal aorta. Although multiple renal arteries were found in human [8, 9] and dog [3, 14], we found that a single renal artery was found in 100% of evaluated rat kidneys, as reported by authors in pig [7], rat [2], rabbit [19], cat [6], sheep [18], and wolf [30].

We noticed that the primary division was a dorsal (D) and a ventral (V) branch of renal arteries. This finding has been reported in some mammals [2, 11, 30]. But, some researchers also found that the primary division of the pig renal artery was a cranial and a caudal branch in 93.4% of cases [7]. Moreover, in human, if there is only one renal artery, the primary division was divided into two branches called as anterior and posterior branches [21].

As reported by the author, in rat [2] and dog [11] cases, the dorsal and ventral branches of renal arteries were divided into two segmental arteries, known as a cranial and a caudal branch. Then, the cranial and caudal branch of renal arteries gave off the interlobar arteries. However, some authors stated that in calve [20], rabbit [19], goat [18], sheep [4], and wolf [30], the dorsal and ventral branch of renal arteries directly gave off the interlobar arteries. However 4-5 segmental branches were found in cat [2, 6] and in human [1, 22]. Moreover, in porcine kidney, it was observed that the renal artery branches were divided into upper and lower polar arteries, and each of these were divided into anterior and posterior segmental arteries [16]. They found that the branching pattern of the pig renal artery was different from the human renal artery [1, 16].

Although left renal artery was reported to be longer than right [6], some researchers found the opposite of this finding in some species [4, 14, 23]. In all cases, in investigation, right renal artery was slightly longer than the left renal artery, because of the aorta's location to the left of the middle plane of the abdominal cavity as human.

Graves [1] was the first person who recognized the five renovascular segments in the human kidneys. Kher et al. [31] modified the grouping of the Graves [1]. SYKES [24] described the venous type of branching of the renal artery and classified that renal arteries were divided into three types, according to their made of branching.

In the present study, we utilized from the study of Marques-Sampaio et al. [11] on dogs' renal arteries branching in order to classify different segmental branching of renal arteries (Table 1). Marques-Sampaio et al. [11] separated the renal arteries branching into six types, whereas we classified it in our study into just 5 types (Table 3). Moreover, in monkey, these arteries were classified into four types [25]. The first and second of these types were similar to type Ia and type Ib which were described by us. Marques-Sampaio et al. [11] presented Type Ie formed by two extra branches running the caudal pole. But this type was not found in the present study.

In this study, the origin of primer branching of renal arteries of rats was found to be more regular than human [8, 9], pig [7], and dog [11, 14]. What is more important, is that renal arteries of SD were more regular than W. Therefore, these finding would support the use of the SD as an animal model for urologic procedures in the dorsal and ventral surface kidney.

In previous studies, the sexual dimorphism of rat kidney morphology was demonstrated [32, 33]. In present study, it was shown that the male kidneys were heavier than those of the female. This finding was the same as the ones obtained from animals like camel [34], rat, and mouse [35, 36]. In this study, we noticed that there was not any effect of kidney and rat weights on the number of the segmental arteries and interlobar arteries of both sexes, except the length of renal arteries.

It is hoped that the results of the present study will encourage further research in this field.

## Figures and Tables

**Table 1:** Comparative morphometric values of the kidney in Wistar and Sprague-Dawley rat (mean ± SEM).

	SD male (*n* = 14)	SD female (*n* = 14)	All SD (*n* = 28)	W male (*n* = 14)	W female (*n* = 14)	All W (*n* = 28)
Live weight	279.5 ± 26.18	237.0 ± 19.7	258.26 ± 5.9	192.9 ± 19.05	171.93 ± 12.4	182.4 ± 19.05
Right kidney weight	1.218 ± 0.11	1.01 ± 0.064	1.11 ± 0.026	0.817 ± 0.110	0.747 ± 0.065	0.782 ± 0.095
Left kidney weight	1.16 ± 0.096	0.964 ± 0.113	1.06 ± 0.027	0.799 ± 0.105	0.726 ± 0.075	0.763 ± 0.097

**Table 2:** Comparative morphometric values of the renal arteries in Wistar and Sprague-Dawley rat (mean ± SEM).

	SD male (*n* = 14)	SD female (*n* = 14)	All SD (*n* = 28)	W male (*n* = 14)	W female (*n* = 14)	All W (*n* = 28)
A1	1.098 ± 0.191	0.902 ± 0.313	1.0 ± 0.0287	0.931 ± 0.043	0.813 ± 0.031	0.872 ± 0.029
D1	0.516 ± 0.026	0.458 ± 0.016	0.480 ± 0.02	0.422 ± 0199	0.449 ± 0.014	0.435 ± 0.012
V1	0.477 ± 0.013	0.414 ± 0.014	0.450 ± 0.012	0.456 ± 0.0132	0.397 ± 0.0117	0.426 ± 0.010
Db1	5.200 ± 0.133	5.400 ± 0.305	5.300 ± 0.163	5.3 ± 0.15	5.3 ± 0.15	5.3 ± 0.10
Vb1	6.20 ± 0.13	6.00 ± 00	6.10 ± 0.068	6.3 ± 0.21	6.2 ± 0.13	6.2 ± 0.12
A2	0.998 ± 0.025	0.801 ± 0.039	0.899 ± 0.032	0.878 ± 0.133	0.799 ± 0.074	0.838 ± 0.112
D2	0.527 ± 0.021	0.467 ± 0.015	0.491 ± 0.014	0.503 ± 0.021	0.470 ± 0.23	0.486 ± 0.015
V2	0.55 ± 0.009	0.479 ± 0.012	0.516 ± 0.011	0.563 ± 0.023	0.469 ± 0.024	0.516 ± 0.019
Db2	5.40 ± 0.22	5.70 ± 0.30	5.550 ± 0.184	5.4 ± 0.221	5.3 ± 0.15	5.3 ± 0.1
Vb2	6.10 ± 0.3	6.1 ± 0.1	6.050 ± 0.05	6.2 ± 0.13	6.2 ± 0.13	6.2 ± 0.09

A1: the length of root of right renal artery; A2: the length of root of left renal artery; D1: the length of the dorsal branch of right renal artery; D2: the length of the dorsal branch of left renal artery; V1: the length of the ventral branch of right renal artery; V2: the length of the ventral branch of left renal artery; Db1, Vb1, Db2, DV2: the number of interlobar arteries of the dorsal and ventral branch of right renal artery and left ones, respectively.

**Table 3:** Shows the study carried out by Marques-Sampaio et al. (2007) [11] to study the variations of the dorsal division (Dd) and ventral division (Vd) of the renal artery and their respective findings were compared to the present study.

	Marques-Sampaio et al., (2007) [11]		Present study			
			W		SD	
Total of kidneys studied	**92**		**56**		**56**	

Types	Dd	Vd	Dd	Vd	Dd	Vd

Type Ia	64.2%	67.3%	30.3	25	17.9	24.1
Type Ib	25.2%	21%	55.5	64.3	60.8	53.7
Type Ic	3.2%	6.3%	3.6	0	0	1.7
Type Id	1.1%	1.1%	8.9	10.7	17.8	17.8
Type Ie	0	3.2%	0	0	0	0
Type II	6.3%	3.2	1.7	0	3.5	1.7
